# Improving hybrid breeding efficiency in winter oilseed rape under sparse multi-environment testing

**DOI:** 10.1007/s00122-026-05341-3

**Published:** 2026-08-12

**Authors:** Lars Erik Thomsen, Matthias Frisch, Christian Flachenecker, Amine Abbadi, Tobias Kox, Cyriakus Kulle, Carola Zenke-Philippi

**Affiliations:** 1 Institute of Agronomy and Plant Breeding II, Justus-Liebig-University Giessen, Heinrich-Buff-Ring 26, 35392 Giessen, Germany; 2 NPZ Innovation GmbH, Hohenlieth Hof 1, 24363 Holtsee, Germany; 3 Norddeutsche Pflanzenzucht Hans-Georg Lembke KG, Hohenlieth Hof 1, 24363 Holtsee, Germany

## Abstract

**Key message:**

Balanced, relationship-based omission, multi-environment predictions, and pedigree-genomic matrices improve genomic predictions, highlighting breeder’s ability to influence performance through strategic training set design and model choice.

**Abstract:**

Genomic prediction is increasingly applied in plant breeding, yet its robustness under sparse testing in hybrid breeding programs of winter oilseed rape (*Brassica napus* L.) lacks evidence. We evaluated the effects of data omission strategies, prediction schemes and models on genomic prediction accuracy and selection consistency. Seven omission scenarios were assessed for three traits with contrasting genetic architectures, using the correlation between observed and predicted phenotypes and Cohen’s *κ* as complementary metrics for genomic prediction accuracy and selection consistency. Both declined systematically with increased data omission, although both metrics only correlated moderately. Balanced omission across environments outperformed unbalanced strategies by stabilizing variance estimation and preserving heritability. Our relationship-informed omission strategy further improved prediction performance, yielding higher and more stable prediction accuracies. Models based on combined pedigree–genomic relationship matrices enabled the inclusion of non-genotyped parental lines and further improved prediction performance. Multi-environment predictions using GCA + SCA models were consistently superior to per-environment predictions and GCA-only models; however, responses were trait-specific. Overall, our results demonstrate that informed omission strategies and model choices substantially influence the performance of genomic prediction under data reduction. Relationship-based balanced omission represents a promising selection approach that balances prediction performance with resource efficiency.

**Supplementary Information:**

The online version contains supplementary material available at https://doi.org/10.1007/s00122-026-05341-3.

## Introduction

The global demand for suitable agricultural practices and high-yielding crop varieties has intensified hybrid breeding (Anbarasan and Ramesh 2022). In this context, oilseed rape (*Brassica napus* L.) is a major crop, accounting for approximately 13% of edible oil production worldwide and 35% in Europe (FAOSTAT 2025). The number of field plots per individual in phenotyping strongly influences the accuracy in selecting superior candidates (Cobb et al. 2019). However, high costs associated with extensive field trials (Burud et al. 2017; Reynolds et al. 2019) contribute a major limitation to the scale of practical breeding programs. At the same time, declining costs for generating genotypic profiles (McCouch et al. 2020; Tripodi 2023) enable broad characterizations of breeding material, adding profound information that complements phenotypic data and enhances the efficiency of selection decisions.

This development has empowered the integration of genomic predictions into plant breeding, offering the potential to increase selection gain per time unit (Falconer and Mackay 1996) while lowering phenotyping costs and to transform conventional plant breeding strategies (Heffner et al. 2009; Jannink et al. 2010). In genomic prediction, genome-wide marker data are utilized to predict the performance of non-phenotyped genotypes (Meuwissen et al. 2001; VanRaden 2008), using a representative training population. Beyond predicting the performance of completely untested genotypes, reducing the number of field plots within the training population can lower phenotyping requirements even further through the application of sparse testing (Jarquin et al. 2020; Atanda et al. 2021, 2022; Montesinos-López et al. 2023). In simulations of unreplicated field trials, genomic predictions have been shown to provide precise estimates of phenotypic values, with correlations to true genotypic values often surpassing those obtained from phenotypic values (Terraillon et al. 2022). This suggests that genomic predictions can achieve similar or even superior accuracy compared to phenotypic values derived from replicated multi-environmental field trials (MET), offering a pathway to reduce phenotyping efforts (Terraillon et al. 2022).

However, with fewer phenotypic observations, key parameters that directly affect prediction accuracy are altered, including trait heritability (Heffner et al. 2009; Combs and Bernardo 2013), the size of the training population (Isidro et al. 2015; Akdemir and Isidro-Sánchez 2019; Edwards et al. 2019), and the relatedness of training and test population (Lozada et al. 2019; Alemu et al. 2023). In contrast, parameters such as population structure (Riedelsheimer et al. 2013; Rincent et al. 2017; Werner et al. 2020) and marker density (Hickey et al. 2014; Norman et al. 2018) remain constant in the context of sparse testing.

While these aspects highlight the potential of sparse testing to reduce costs, they also underscore the challenges of designing training populations that preserve high prediction accuracy under reduced phenotyping. Previous studies have demonstrated that the success of sparse testing depends on the strategy of omission applied and the genomic prediction scheme used (Jarquin et al. 2020; Crespo-Herrera et al. 2021; Lell et al. 2021; Atanda et al. 2022), indicating that inappropriate omission can rapidly erode prediction performance. In commercial wheat breeding programs, Thomsen et al. (2026) showed that larger training populations cannot compensate for low phenotypic data quality, highlighting the need to balance data quantity and reliability in sparse testing. Furthermore, evidence for winter oilseed rape (WOSR) remains limited, particularly regarding the impact of performing genomic prediction at the environment level versus multi-environment phenotypes and the interaction between omission strategies and prediction models. Moreover, although relatedness between genotypes in the training set and the predicted test set is known to play a central role in genomic predictions (Lozada et al. 2019; Alemu et al. 2023), its explicit use to guide data omission and sustain prediction performance has received little attention in sparse testing, with approaches often relying on structured but implicit preservation of family connectivity (Lell et al. 2021). Moving from implicitly preserved to explicitly exploited relatedness of predicted genotypes to the training set is a promising way for enhancing genomic prediction in hybrid breeding and for aligning the efficiency advantages of sparse testing with robust predictive performance. In this context, our study aims to advance strategies that enhance the efficiency of genomic prediction in a WOSR hybrid breeding program.

The objectives of our study were to investigate the influence of (i) different omission scenarios across a range of omission rates with (ii) different data aggregation levels and (iii) prediction models with and without specific combining ability (SCA) effects on the genomic prediction performance of three traits in 4,000 WOSR hybrids.

## Material and methods

### Plant material

The germplasm analyzed in this study was developed through the WOSR breeding program of the commercial breeding company of Norddeutsche Pflanzenzucht Hans-Georg Lembke KG (NPZ). The genotypes are elite hybrids selected for their suitability for grain yield and oil production in Central Europe.

Phenotypic data for a total of 4,000 hybrids evaluated between 2022 and 2025 were obtained from 61 METs implemented in alpha lattice design without replications. Each MET included 72 hybrids with three check varieties being common across METs and was cultivated at five to seven locations, collectively covering 31 distinct environments, defined as combinations of locations and years (Supplementary Information (SI) 1). For the subsequent analyses, each MET was treated as a separate dataset, reflecting the practical structure of breeding programs where METs are often analyzed independently. For each MET, two environments conducted at locations operated directly by the breeder were classified as internal environments, whereas other environments conducted at partner or externally managed locations were classified as external environments. The phenotypic data contained a total of 27,360 plots, managed with the application of fertilizers, fungicides and insecticides, and with sizes varying from 9.75 m^2^ to 13.5 m^2^. Records were available for grain yield (YSD), grain oil content (OIL), and meal protein content (PRM).

Grain yield was determined by weighting the harvested grain using a combine harvester, with the recorded yield representing raw yield prior to post-harvest adjustments. A 100 g seed sample was collected from each field plot during harvest and analyzed for seed moisture content, grain oil content, and grain protein content with near-infrared reflection spectroscopy (Polytec GmbH, Model ZilvA V 2.0, Waldbronn, Germany). Seed moisture content was used to adjust the weight of the harvested grain to a relative moisture content of 9.0%. These corrected YSD values were then expressed in quintal per hectare (q/ha^−1^). OIL was likewise corrected for seed moisture content. Measured grain protein content was transformed to PRM by using a standard conversion factor that adjusts for oil removal and associated compositional changes during processing.

Genomic profiles for 1,284 of 1,724 parental lines were generated using a 60 K single-nucleotide polymorphism (SNP) array, yielding in 27,214 polymorphic SNPs for use in the present study. The homozygous parental inbred lines formed a factorial with no within-group hybrids. Per set of 72 hybrids of a MET, an average of 12.4 mother lines (range 2–40) and 34.4 father lines (range 15–61) were represented. This design realized 1.67% of all possible combinations between mother and father lines and 26.3 hybrids on average per mother line. SNP profiles for hybrids were derived from the information of SNP profiles of the respective homozygous parental inbred lines. To characterize the population structure within the germplasm, Rogers’ distances (Rogers 1972) were calculated for all pairs of parental lines, followed by a principal coordinate analysis (PCoA).

### Omission scenarios

**Fig. 1:**
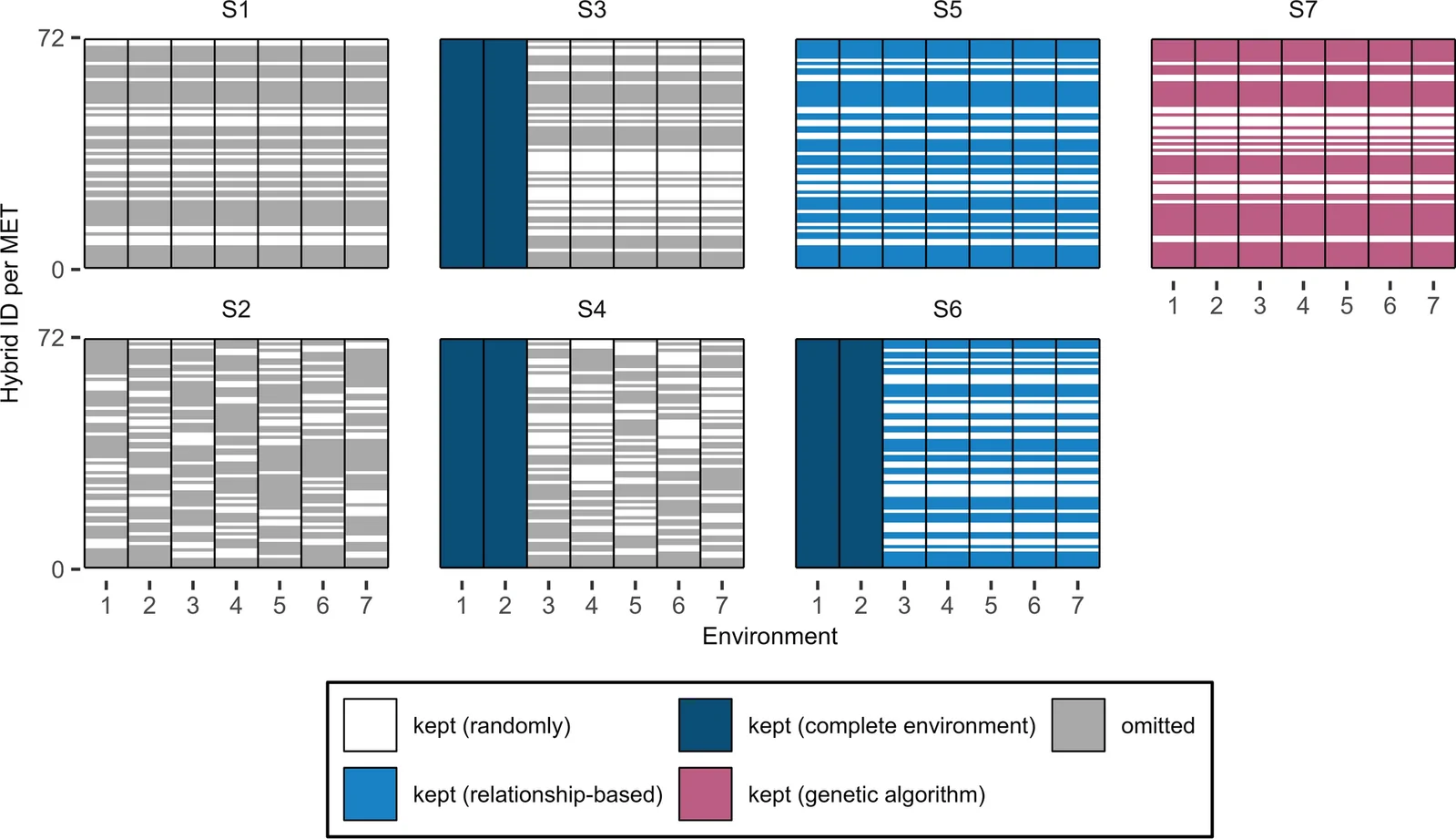
Schematic illustration of omission scenarios (S1-S7) for a targeted proportion of data reduction of 30% in a multi-environment field trial (MET) compiling 72 hybrids

Seven in silico scenarios were defined to assess the robustness of different prediction models through simulating the masking of phenotypic data by omitting a proportion of phenotypic data independently in each MET (Fig. 1). This ensures that the sparseness introduced by the omission scenarios aligns with the structure of the data. Omission rates of 0%, 10%, 20%, 30%, 40%, and 50% were applied across all scenarios. Check varieties in all METs were retained in all cases to preserve cross-environment comparability, and omissions were applied exclusively to non-check entries.

The first four omission scenarios were conceptualized to investigate how different patterns of data reduction affect the prediction performance, focusing on the influence of omission uniformity and the extent of entry overlap across environments. To capture variability introduced by random sampling, the first four scenarios were repeated for 25 independent cross-validation replicates. These scenarios were defined as follows.

- S1: A random sample of the non-check entries within a MET was omitted uniformly across all environments. Thus, if a given hybrid was removed in one environment, it was also absent in every other environment of that MET. This setup reflected situations where specific entries are not tested in any environment, and the proportion of omitted entries was consistent across environments.
- S2: The random set of omitted entries differed independently between environments within a given MET. Consequently, the specific entries omitted in one environment were not the same as those omitted in other environments. Across the MET, all entries remained represented with phenotypic data in at least one environment. The number of omitted plots was set to identical values for each environment to achieve a uniform percentage of data reduction.
- S3: The environments present in METs were split into breeder´s environments (internal sites) and external environments. The complete set of entries were retained in internal environments, ensuring complete datasets and guaranteeing overlap in all entries across these environments. The number of omitted plots in random samples of non-check entries was omitted uniformly across all remaining external environments such that the total proportion of omitted plots across the entire MET matched the targeted level.
- S4: Similar to S3, environments within each MET were divided into internal and external environments, with all entries retained at internal environments to ensure complete datasets and consistent overlap across these environments. In external environments, however, the omission of non-check entries was conducted independently, meaning that different random subsets of entries were removed in each external environment. The number of omitted plots in external environments was adjusted such that when aggregated across the entire MET, the proportion of omitted plots matched the targeted level.

For scenarios S5 and S6, a novel deterministic omission method was implemented, replacing random removal with a systematic, relationship-based strategy to iteratively select hybrids for exclusion. Specifically, hybrids were sequentially removed from the training set based on their average relationship coefficient to the remaining training population, with the hybrid exhibiting the highest average relationship coefficient being omitted at each iteration until the predefined number of hybrids was reached, as:

1 $$O = \bigcup\limits_{t = 1}^{{n_{{{\mathrm{omit}}}} }} {\left\{ {{\mathrm{arg}}\mathop {\max }\limits_{{x \in H\backslash O_{t - 1} }} \left( {\frac{1}{{\left| {H\backslash \left( {O_{t - 1} \bigcup {\left\{ x \right\}} } \right)} \right|}} \mathop \sum \limits_{{y \in H\left( {O_{t - 1} \bigcup {\left\{ x \right\}} } \right)}} r_{xy} } \right)} \right\}} , O_{0} = \emptyset ,$$

where O is the cumulative set of hybrids selected for omission across all iterations, ${O}_{0}$ denotes the empty set representing that no hybrids have been omitted before the first iteration, ${O}_{t-1}$ is the set of hybrids already omitted in the previous iteration, H is the total set of hybrids included in a MET, ${r}_{xy}$ the relationship coefficient between hybrid x and y, and ${n}_{\mathrm{o}\mathrm{m}\mathrm{i}\mathrm{t}}$ is the targeted number of hybrids to omit per MET, which is either applied uniformly across all environments (S5) or adjusted to meet the targeted proportion of omission when aggregated across all environments of the MET (S6). The union operator $\bigcup \left\{x\right\}$) denotes the combination of sets, such that ${O}_{t-1}\bigcup \left\{x\right\}$ represents the previously omitted hybrids together with candidate hybrid x. The set difference operator “\” denotes the exclusion of elements from a set, thus $H\backslash \left({O}_{t-1}\right)$ represents all hybrids remaining after previous omission, whereas $H\backslash \left({O}_{t-1}\right)\bigcup \left\{x\right\}$ denotes the remaining hybrids after additionally excluding candidate hybrid x. The relationship coefficient ${r}_{xy}$ was calculated by combining a relationship matrix (Eq. 4) derived from reconstructed SNP data of the hybrids, which were inferred based on the SNP profiles of their parental lines, with available pedigree information. This computation was carried out using the package AGHmatrix (v2.1.4; Amadeu et al. 2023) in R Statistical Software (v4.4.3; R Core Team 2025). The goal of this deterministic procedure is to iteratively identify the hybrid whose removal maximizes its mean relatedness to the remaining training set (SI 2a), thereby retaining genetically representative training sets and preserving connectivity between the retained training set and the test set. As this procedure was fully deterministic and involved no random components, only a single realization was required. Two deterministic omission scenarios were defined as:

- S5: Relationship-based omission was applied uniformly across all environments of a MET. Non-check entries were selected for omission based on their mean genetic relatedness to the remaining training set, such that the same hybrids were omitted in every environment and the proportion of omitted entries was consistent across environments.
- S6: The same relationship-based omission strategy was used as in S5, but omission of non-check hybrids was restricted to external environments only. All hybrids were retained in internal environments to ensure full entry overlap, while the number of omitted plots in external environments was adjusted so that the targeted proportion of omission was achieved across the entire MET.

Finally, a genetic algorithm-based training set optimization was implemented using the package STPGA (v5.2.1; Akdemir 2017) in R Statistical Software (v4.4.3; R Core Team 2025). The training set was optimized using the CDMEAN criterion based on the same hybrid relationship matrix (Eq. 4 applying derived SNPs and pedigree information) used in S5 and S6. For each optimization, the genetic algorithm was run with a maximum of 100 iterations, a population size of 50, five elite solutions, a mutation probability of 0.5, a mutation intensity of 1, and a scalar shrinkage parameter of 1 × 10^−9^. All other parameters were kept at their default values. A single optimization for each MET and model configuration was performed to provide a computationally efficient workflow representative of practical breeding applications.

- S7: The genetic algorithm selects an optimized subset of non-check hybrids for the training set, and the remaining hybrids were omitted (SI 2b). Omission was applied uniformly across all environments within each MET.

### Prediction schemes

**Fig. 2:**
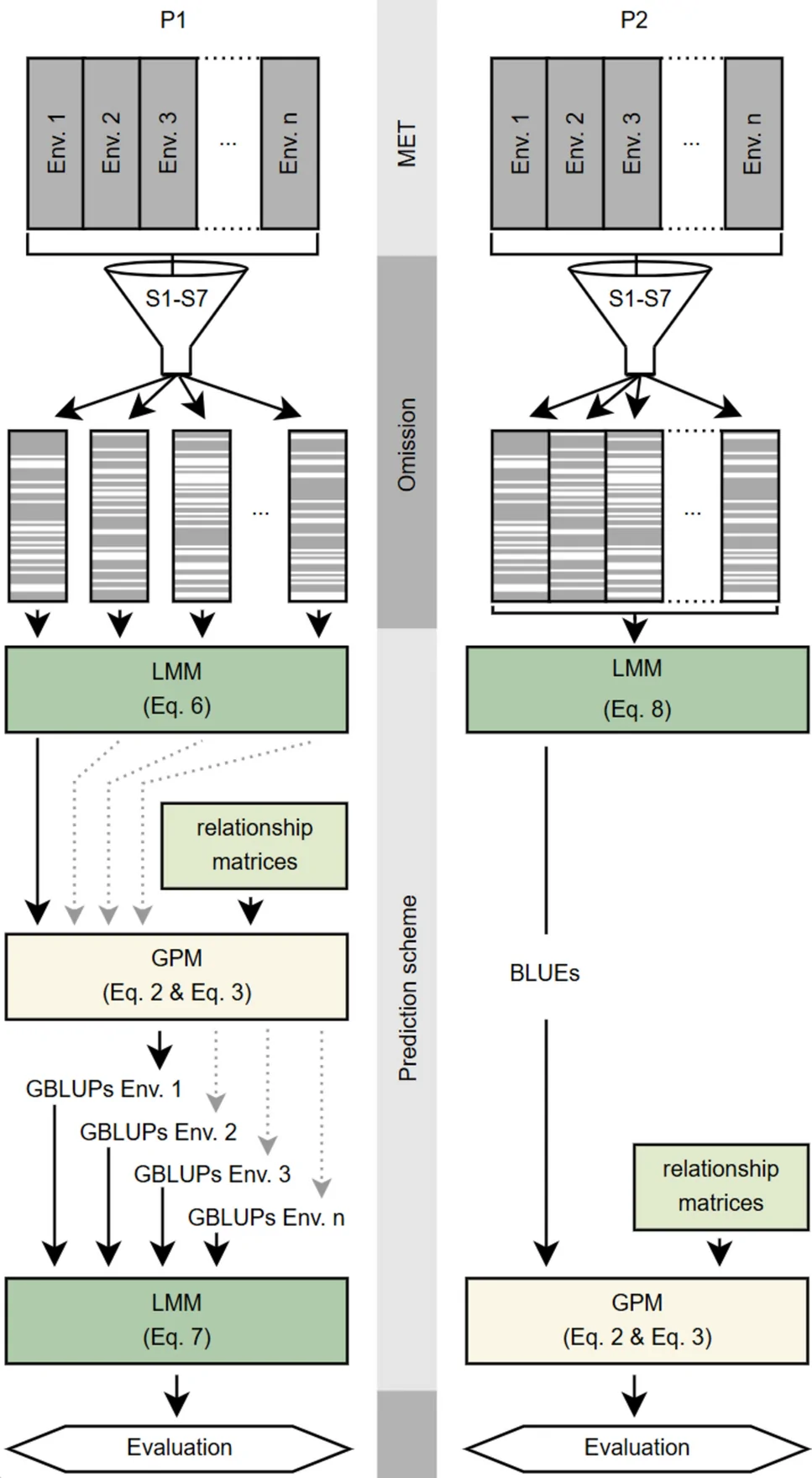
Schematic illustration of two prediction schemes (P1 = per-environment predictions, P2 = complete-MET predictions) performed on each multi-environmental field trial (MET) after reducing data according to one omission scheme (S1-S7). Env. = environment, GPM = genomic prediction model, LMM = linear mixed model, GBLUPs = genomic best linear unbiased predictions, BLUEs = best linear unbiased estimators, Eq. = equation

After the omission of phenotypic data, two prediction schemes were evaluated to assess the effect of data aggregation across environments on the prediction performance (Fig. 2). For per-environment predictions (P1), phenotypic data from each environment within the MET were processed independently to generate environment-specific predictions. These predictions were subsequently merged across all environments of the MET to obtain the final estimate for each hybrid. For complete-MET predictions (P2), each MET was likewise analyzed separately; however, adjusted means were first derived from phenotypic data of all environments of the MET, which then served as the input for genomic predictions.

In each prediction scheme, two genomic prediction models were applied, both incorporating the general combining ability (GCA) effects for female and male parents, but differing in the inclusion of specific combining ability (SCA) effects for parental combinations (Eqs. 2 and 3):

2 $$\mathit{y}_{{{\mathit{fm}}}} = \mu_{0} + \mathit{Z}_{{\mathit{f}}} {\mathit{GCA}}_{{\mathit{f}}} + \mathit{Z}_{{\mathit{m}}} {\mathit{GCA}}_{{\mathit{m}}} + \mathit{\varepsilon}_{{{\mathit{fm}}}}$$

3 $${\mathit{y}_{{\mathit{fm}}}} = \mu_{0} + \mathit{Z}_{{\mathit{f}}} {\mathit{GCA}}_{{\mathit{f}}} + \mathit{Z}_{{\mathit{m}}} {\mathit{GCA}}_{{\mathit{m}}} + \mathit{Z}_{{{\mathit{fm}}}} {\mathit{SCA}}_{{{\mathit{fm}}}} + \mathit{\varepsilon}_{{{\mathit{fm}}}}$$

Here, $\mathit{y}_{\mathit{fm}}$ denotes the vector of phenotypic values of the hybrids for each MET, and ${\mu}_{0}$ is the fixed model intercept. The matrices $\mathit{Z}_{\mathit{f}}$ and $\mathit{Z}_{\mathit{m}}$ are the design matrices linking the random GCA effects of the female and male parents, respectively, with their hybrid progeny, whereas $\mathit{Z}_{\mathit{fm}}$ links the SCA effects to the hybrids. The GCA effects ${\mathit{GCA}}_{\mathit{f}}$, ${\mathit{GCA}}_{\mathit{m}}$, and SCA effects ${\mathit{SCA}}_{\mathit{fm}}$, were assumed to follow multivariate normal distributions with covariance matrices $\mathit{G}_{\mathit{f}}{\sigma}_{{\mathit{GCA}}_{\mathit{f}}}^{2}$, $\mathit{G}_{\mathit{m}}{\sigma}_{{\mathit{GCA}}_{\mathit{m}}}^{2}$, and $\mathit{G}_{\mathit{fm}}{\sigma}_{{\mathit{SCA}}_{\mathit{fm}}}^{2}$, respectively. ${\sigma}_{{\mathit{GCA}}_{\mathit{f}}}^{2}$, ${\sigma}_{{\mathit{GCA}}_{\mathit{m}}}^{2}$, and ${\sigma}_{{\mathit{SCA}}_{\mathit{fm}}}^{2}$ were the variance components being pertin7ent to GCA and SCA effects of all observed hybrids in the factorial f×m of a MET. $\mathit{G}_{\mathit{fm}}$ refers to the Kronecker product of $\mathit{G}_{\mathit{f}}\otimes \mathit{G}_{\mathit{m}}$ (Stuber and Cockerham 1966), where $\mathit{G}_{\mathit{f}}$ and $\mathit{G}_{\mathit{m}}$ denote relationship matrices constructed as genomic relationship matrices ($\mathit{G}_{\mathit{f}}^{(G)}$ and $\mathit{G}_{\mathit{m}}^{(G)}$, respectively). These matrices were computed from SNP data subsets $R={(\mathrm{S}\mathrm{N}\mathrm{P}}_{\mathrm{f}}\vee {\mathrm{S}\mathrm{N}\mathrm{P}}_{\mathrm{m}})$ for all pairs of lines (i,j) within the same heterotic group (either f or m), respectively. R refers to ${R}_{\mathit{f}}$ for ${G}_{\mathit{f}}$ and ${R}_{\mathit{m}}$ for ${G}_{\mathit{m}}$, ensuring that the relationship matrices are computed using the SNP subsets specific to each heterotic group. We followed the simplified approach of Schopp et al. (2017), corresponding to Habier et al. (2007) and VanRaden (2008):

4 $$\mathit{G}_{{\mathit{f}}}^{\left( G \right)} \left( {i,j} \right), \mathit{G}_{{\mathit{m}}}^{\left( G \right)} \left( {i,j} \right) = \frac{{\mathop \sum \nolimits_{l \in R} \left( {x_{{i,{\mathit{l}}}} - 2p_{{\mathit{l}}} } \right) \left( {x_{j,l} - 2p_{{\mathit{l}}} } \right)}}{{2\mathop \sum \nolimits_{l \in R} p_{{\mathit{l}}} \left( {1 - p_{{\mathit{l}}} } \right)}} ,$$

where ${x}_{i,l}, {x}_{j,l}\in \left\{0, 1, 2\right\}$ denote the genotypic scores of individuals i and j within heterotic group f or m, respectively, representing the number of reference alleles carried at locus l, and ${p}_{\mathit{l}}$ is the reference allele frequency at locus l within the respective heterotic group f or m from which i and j originated. Alternatively, combined pedigree–genomic relationship matrices ($\mathit{G}_{\mathit{f}}^{(H)}$ and $\mathit{G}_{\mathit{m}}^{(H)}$) were constructed to include not only 1,284 genotyped parental inbred lines but also 440 additional non-genotyped individuals with available pedigree data in the subsequent predictions. To further disentangle whether differences arises from the inclusion of non-genotyped individuals of from the pedigree information itself, an evaluation of ${G}^{(H)}$ using only fully genotyped hybrids was conducted, thus matching the training set size of ${G}^{(G)}$. ${G}^{(H)}$ matrices integrate pedigree-based and genomic information following the original approach of Martini et al. (2018) using the package AGHmatrix (v2.1.4; Amadeu et al. 2023) in R (v4.4.3; R Core Team 2025). To ensure comparability, all relationship matrices were normalized to unit average diagonal (Xu 2013).

Exemplarily, the GCA effects in heterotic group f were predicted using $\hat{g}_{{\mathrm{f}}} = \sigma_{{{\mathit{GCA}}_{{\mathit{f}}} }}^{2} \mathit{G}_{{\mathit{f}}} \mathit{Z}_{{\mathit{f}}}{\prime} \mathit{V}_{{{\mathit{fm,}}\;{\mathit{fm}}}}^{ - 1} \mathit{y}_{{{\mathit{fm}}}}$, where $\mathit{G}_{\mathit{f}}$ represents the relationship matrix among individuals in f, $\mathit{Z}_{\mathit{f}}{\prime}$ is the transpose of the design matrix linking hybrid observations to their respective female parents, $\mathit{V}_{\mathit{fm},\mathit{fm}}^{-1}$ denotes the inverse of the phenotypic covariance matrix, and $\mathit{y}_{\mathit{fm}}$ is the vector of phenotypic values of the hybrids (Melchinger et al. 2023). ${\sigma}_{{\mathit{GCA}}_{\mathit{f}}}^{2}$, ${\sigma}_{{\mathit{GCA}}_{\mathit{m}}}^{2}$, and ${\sigma}_{{\mathit{SCA}}_{\mathit{fm}}}^{2}$ and the residual error variance (${\sigma}_{\mathit{res}}^{2}$) were extracted from the fitted mixed models of Eq. 3. These estimates were subsequently used to quantify the relative contribution of SCA effects to the sum of genetic effects by computing their ratio expressed as:

5 $$\tau = \frac{{\sigma_{{{\mathit{SCA}}_{{{\mathit{fm}}}} }}^{2} }}{{\sigma_{{\mathit{G}}}^{2} }},$$

where ${\sigma}_{\mathit{G}}^{2}$ represents the total genetic variance, defined as the sum of ${\sigma}_{{\mathit{GCA}}_{\mathit{f}}}^{2}$, ${\sigma}_{{\mathit{GCA}}_{\mathit{m}}}^{2}$, and ${\sigma}_{{\mathit{SCA}}_{\mathit{fm}}}^{2}$.

For prediction scheme P1, response values from each environment of a MET were first corrected for site effects (Fig. 2) using Eq. 6:

6 $$y_{{x{\mathrm{b}}}} = \mu_{0} + g_{x} + b_{{\mathrm{b}}} + e_{{x{\mathrm{b}}}}$$

where ${y}_{xb}$ is the response value hybrid x in block b, ${g}_{x}$ is the genotypic value of hybrid x, ${b}_{b}$ is the block effect of block b, and ${e}_{xb}$ is the residual error term. Best linear unbiased estimates (BLUEs) of ${g}_{x}$ were extracted from Eq. 6 for each environment of a MET and then served as input values for genomic predictions in P1. These predicted values were then combined across environments of a MET using Eq. 7:

7 $$y_{xk} = \mu_{0} + g_{x} + e_{k} + e|\varepsilon_{xk}$$

where each observation ${y}_{xk}$ is decomposed into the genotypic value ${g}_{x}$ of hybrid x, the environment effect ${e}_{k}$ of environment k, and the environment-specific residual error term ${e|\varepsilon }_{xk}$ for each observation. For prediction scheme P2, response values of all environments of a MET were combined before genomic predictions (Fig. 2) using Eq. 8:

8 $$y_{x\mathit{bk}} = \mu_{0} + g_{x} + e_{k} + \left( {eb} \right)_{{{\mathit{kb}}}} + e|\varepsilon_{{{\mathit{xbk}}}}$$

where ${y}_{\mathit{xbk}}$ is the response value of hybrid x in block b of environment k, ${g}_{x}$ is the genotypic value of hybrid x, ${e}_{k}$ is the environment effect of environment k, ${(eb)}_{\mathit{kb}}$ is the block effect of block b nested in the environment effect of environment k, and ${e|\varepsilon }_{\mathit{xkb}}$ is the environment-specific residual error term. BLUEs of ${g}_{x}$ were extracted from Eq. 8 and used as input values for subsequent genomic predictions in P2. The population mean ${\upmu}_{0}$ and the genotypic values g in Eqs. 6–8 are modeled as fixed and all other effects as random, with $x \sim N(0; I{\sigma}_{x})$ for each x in e, b, and ε. The models Eqs. 2, 3, 6, 7 and 8 were fitted using the package sommer (v4.4.1; Covarrubias-Pazaran 2016) in R (v4.4.3; R Core Team 2025).

The broad-sense heritability (${H}^{2}$) was estimated separately for each MET following the intermediate inference space approach of Piepho and Möhring (2007). Using the variance components extracted from phenotypic model described in Eq. 8, ${H}^{2}$ was calculated as:

9 $$H^{2} = \frac{{\sigma_{{\mathit{g}}}^{2} }}{{\sigma_{{\mathit{g}}}^{2} + \overline{v}/2}},$$

where ${\sigma}_{\mathit{g}}^{2}$ denotes the genotypic variance and $\overline{v }$ is the mean variance of a difference of two BLUEs.

### Evaluation metrics

BLUEs from the full phenotypic dataset were derived using Eq. 8 and treated as true reference values for the downstream analysis. For each omission scenario and prediction scheme, hybrid performance was predicted from sparse phenotypic data as the sum of the estimated intercept ${\widehat{\mu }}_{0}$ and parental GCA effects, with or without SCA effects. For model formulation 1 (Eq. 2), hybrid performance was predicted as

10 $$\hat{y}_{{{\mathit{fm}}}} = \hat{\mu}_{0} + \hat{g}_{{\mathit{f}}} + \hat{g}_{{\mathit{m}}} ,$$

while for model formulation 2 (Eq. 3), SCA effects were additionally incorporated as

11 $$\hat{y}_{{{\mathit{fm}}}} = \hat{\mu}_{0} + \hat{g}_{{\mathit{f}}} + \hat{g}_{{\mathit{m}}} + \hat{s}_{{{\mathit{fm}}}} .$$

Since genomic relationships among parents were used for estimation of effects, the resulting ${\widehat{y}}_{\mathit{fm}}$ from Eqs. 10 and 11 correspond to genomic best linear unbiased predictions (GBLUPs) for hybrid performance. Prediction accuracy was assessed by computing the Pearson correlation coefficient between BLUEs from complete-MET data and GBLUPs from reduced MET data, computed across the complete hybrid set of a MET for all omission scenarios (S1-S7) to ensure consistency in evaluation. As for S2, S3, S4, and S6, the definition of the test set is ambiguous, only for S1, S5, and S7 a meaningful additional computation of a test set prediction accuracy was performed.

As a complementary measure to correlation, Cohen´s *κ* (Cohen 1960) was used to quantify the consistency of hybrid selection, accounting for the agreement beyond chance. Specifically, based on BLUEs and based on GBLUPs, hybrids were selected if their respective predicted value exceeded the empirical 90th percentile of that distribution, representing the upper 10% of hybrid performance. The agreement between those two selections was quantified using

12 $${\upkappa } = \frac{{p_{{\mathrm{o}}} - p_{{\mathrm{e}}} }}{{1 - p_{{\mathrm{e}}} }}$$

where ${p}_{\mathrm{o}}$ denotes the observed proportion of agreement and ${p}_{\mathrm{e}}$ is the expected agreement by chance alone. According to Kuhn and Johnson (2013), *κ* values between 0.3 and 0.5 indicate reasonable agreement. To quantify how changes in prediction accuracy affect Cohen’s *κ*, Cohen’s *κ* was modeled as a smooth function of the prediction accuracy using a generalized additive model. Local derivatives were computed numerically to estimate the sensitivity, and the required increase in prediction accuracy for a 0.01 Cohen’s *κ* improvement was calculated as 0.01 divided by the derivate. Estimates were restricted to the 5th to 95th percentile of prediction accuracy to avoid unreliable values in regions with sparse data of near-zero slopes.

Figures were generated in R using the package ggplot2 (Wickham 2016), while Fig. 2 is created with the diagramming software draw.io (2025).

## Results

The PCoA revealed distinct genetic pools reflecting established heterotic groups in hybrid breeding germplasm, with no additional structure expected to influence genomic prediction or the representation of the germplasm in the relationship matrices (SI 3).

**Fig. 3:**
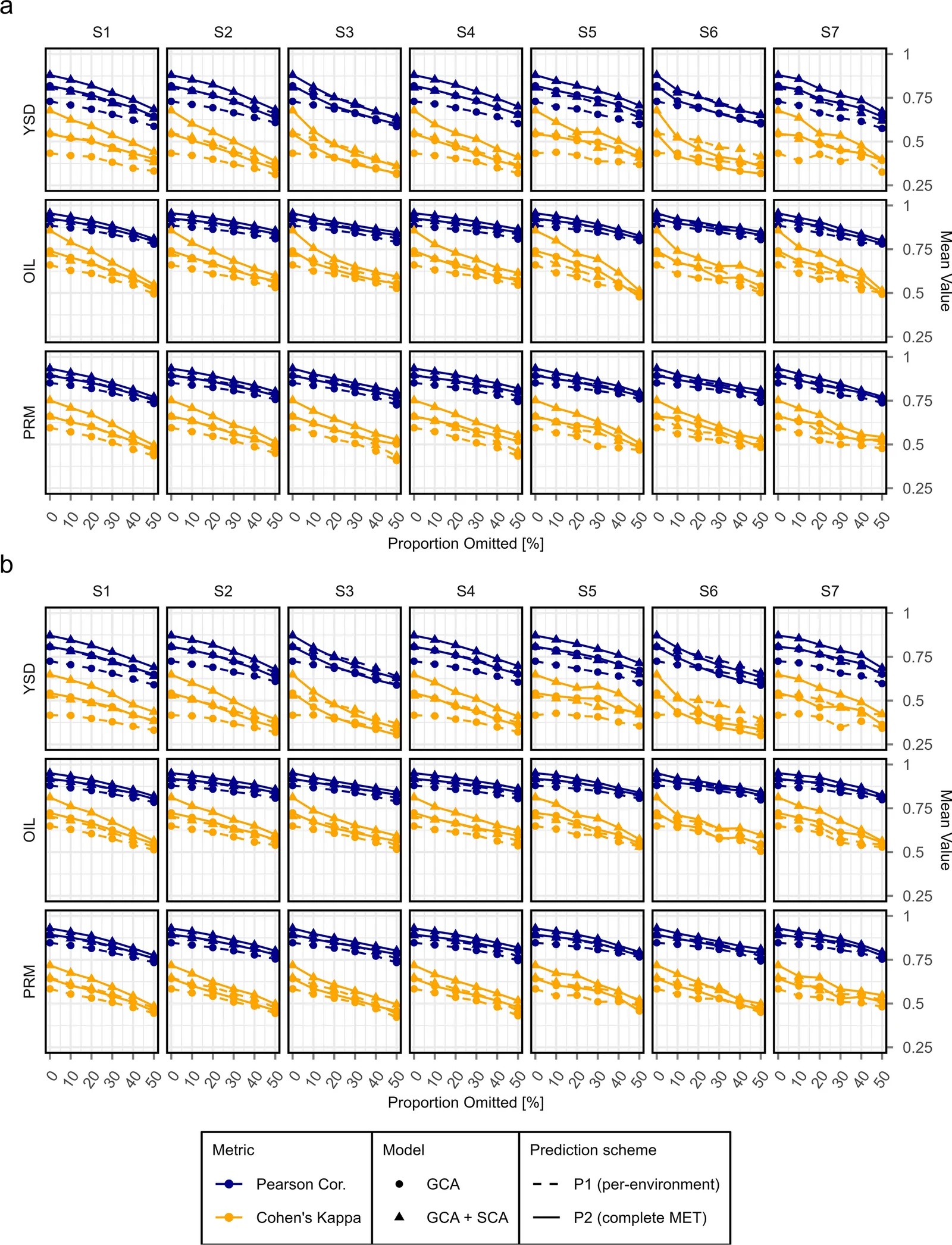
Evaluation metrics for prediction accuracy (Pearson correlation, blue) and selection consistency (Cohen’s *κ*, orange) attained across levels of targeted omission rates in seven omission scenarios (S1-S7) for three traits (YSD = grain yield, OIL = oil content, and PRM = protein content in meal). Results are shown for two prediction models (GCA = circles, and GCA + SCA = triangles) and two prediction schemes (per-environment (P1) = dashed lines, and complete-MET (P2) = solid lines). Panel **a** displays results using genomic relationship matrices (${G}^{(G)}$), whereas panel **b** shows results using pedigree-genomic relationship matrices (${G}^{(H)}$). Values of evaluation metrics represent means across all iterations and METs

Across all omission scenarios (S1-S7), prediction schemes, models, relationship matrices, and traits, both prediction accuracy and Cohen’s *κ* declined systematically with increasing proportions of omitted phenotypic records (Fig. 3). Prediction accuracy and Cohen’s *κ* were moderately correlated (*r* = 0.62) overall, indicating the reduced prediction accuracy came along with lower consistency in hybrid selection. Within the observed range, an increase of 0.01 Cohen’s *κ* required on average a 0.0103 increase in the prediction accuracy, with local slopes ranging from 0.0048 to 0.0145. Declines in Cohen’s *κ* were more pronounced at higher omission rates, particular for complete-MET predictions (P2) (Fig. 3).

Results obtained using ${G}^{(H)}$ (Fig. 3b) closely mirrored those obtained using ${G}^{(G)}$ (Fig. 3a). The mean prediction accuracy for both relationship matrices was similar for the best-performing model combination with prediction scheme P2 and the GCA + SCA model at 0% omission (0.88, 0.95, and 0.93, respectively for YSD, OIL, and PRM). Despite similar prediction accuracies, mean Cohen’s *κ* values using ${G}^{(G)}$ (0.67, 0.85, and 0.75) were slightly higher than with ${G}^{(H)}$ (0.65, 0.81, and 0.72, respectively for YSD, OIL, and PRM) at 0% omission. The similarity in prediction performance obtained using ${G}^{(H)}$ or ${G}^{(G)}$ remained robust even as omission rates increased (Fig. 3).

Differences in prediction performance among omission scenarios were generally modest but trait-dependent. For OIL and PRM, the scenarios produced largely similar outcomes, indicating limited sensitivity to the omission scenario for those traits. In contrast, YSD exhibited greater sensitivity. Scenarios retaining complete internal environments while omitting uniformly among external environments (S3 and S6) showed larger reductions in both prediction accuracy and Cohen’s *κ*, with losses already evident at smaller omission rates. At maximum omission rate (50%) using the GCA + SCA model in prediction scheme P2, the mean prediction accuracies were 0.63, 0.84, and 0.80 and the mean Cohen’s *κ* 0.34, 0.60, and 0.50, for YSD, OIL, and PRM, respectively. Among scenarios with uniform omission across all environments (S1, S5 and S7), S5, with the omission based on relatedness, yielded the highest mean values for prediction accuracy (0.72, 0.84, and 0.80) and Cohen’s *κ* (0.46, 0.58, and 0.52) at 50% omission rate (Fig. 3) for YSD, OIL, and PRM, respectively. Compared with independent omission across environments (S2 and S4), S5 showed a small but consistent advantage for YSD, whereas S4 outperformed S5 for OIL and PRM. Overall, omission scenarios had subtle, primarily trait-specific effects without altering general performance trends.

Across all omission scenarios and traits, prediction scheme P2 (complete-MET; solid lines) generally outperformed prediction scheme P1 (per-environment; dashed lines) in both prediction accuracy and selection consistency, but the magnitude of this advantage depended on the omission rate and trait (Fig. 3). For YSD, the superiority of P2 decreased with rising omission rate in most scenarios. However, in scenarios S3 and S6, selection consistency under P2 declined more strongly, becoming inferior to P1 at high omission rates. For OIL and PRM, differences between prediction schemes were less pronounced, and at the highest omission rate (50%) the advantage disappeared. Despite these exceptions at severe data reduction, P2 exhibited overall superior performance across omission scenarios.

The inclusion of SCA effects into the prediction model consistently outperformed the GCA model across all omission scenarios and traits (Fig. 3). However, the gain from including SCA effects was more pronounced for YSD and less distinct for OIL and PRM. For prediction accuracy, the advantage of GCA + SCA over GCA remained essentially constant across omission rates. In contrast, the strong advantage observed for Cohen’s *κ* at low omission rates declined with increasing omission, although GCA + SCA maintained higher selection consistency across all omission scenarios.

**Fig. 4:**
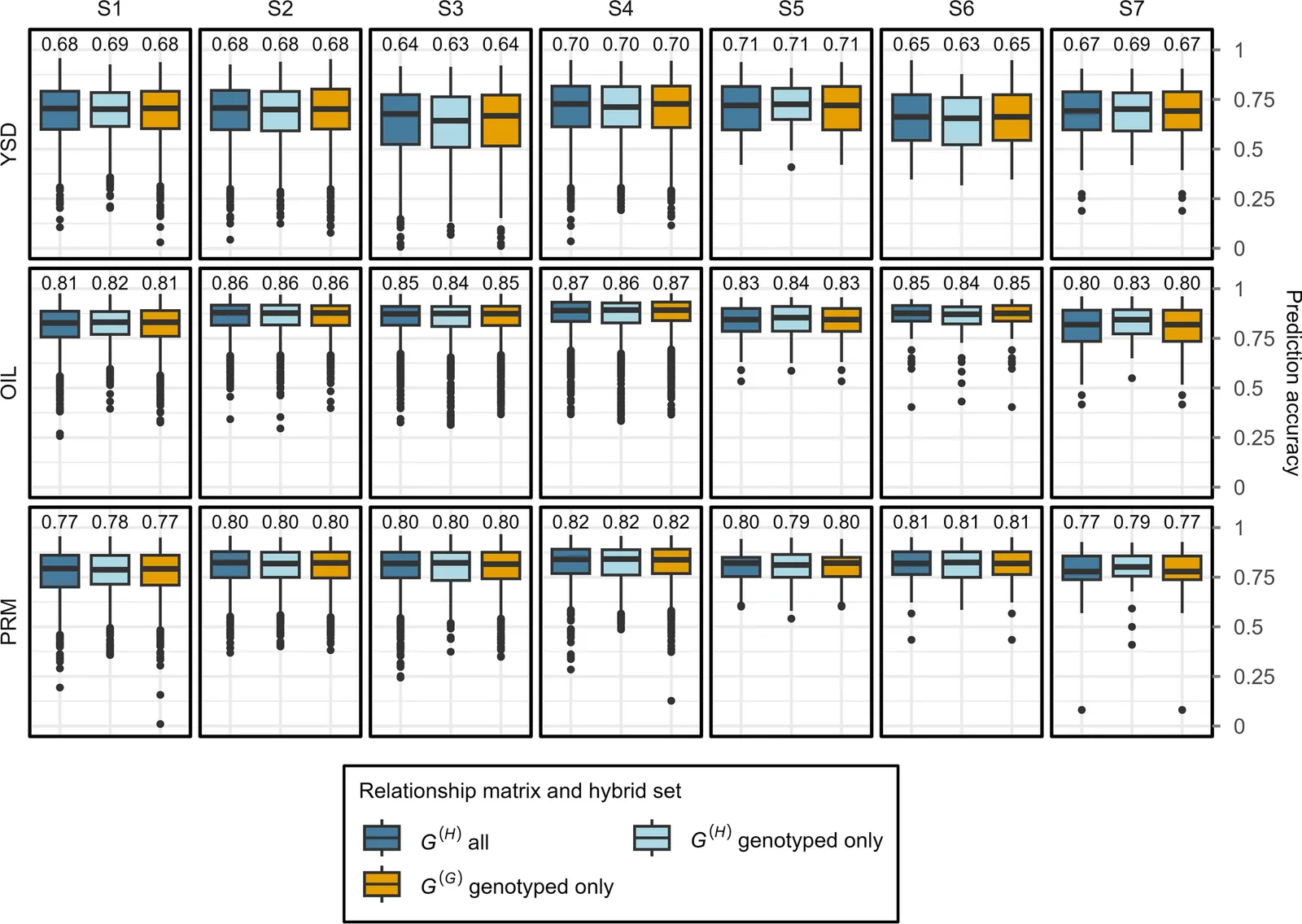
Differences in prediction accuracy for grain yield (YSD), oil content (OIL), and protein content in meal (PRM) across seven omission scenarios (S1-S7) at 50% omission rate, evaluated under the prediction scheme P2 (complete-MET predictions) and GCA + SCA model. Comparisons include genomic relationship matrices ${G}^{(G)}$ and pedigree-genomic relationship matrices ${G}^{(H)}$, with ${G}^{(H)}$ applied to both the complete hybrids sets and the subset of containing only hybrids with genotyped parents. Values of prediction accuracy represent means across all iterations and multi-environment field trials (METs)

To disentangle the respective contributions of training set expansion and pedigree information content, prediction accuracy was evaluated under the best-performing configuration (P2 prediction scheme, GCA + SCA model) at 50% omission rate for all omission scenarios and traits comparing ${G}^{(H)}$ and ${G}^{(G)}$ relationship matrices (Fig. 4). When ${G}^{(H)}$ was applied exclusively to hybrids with genotyped parents, the resulting prediction accuracies were virtually indistinguishable from those obtained with the full hybrid set.

**Fig. 5:**
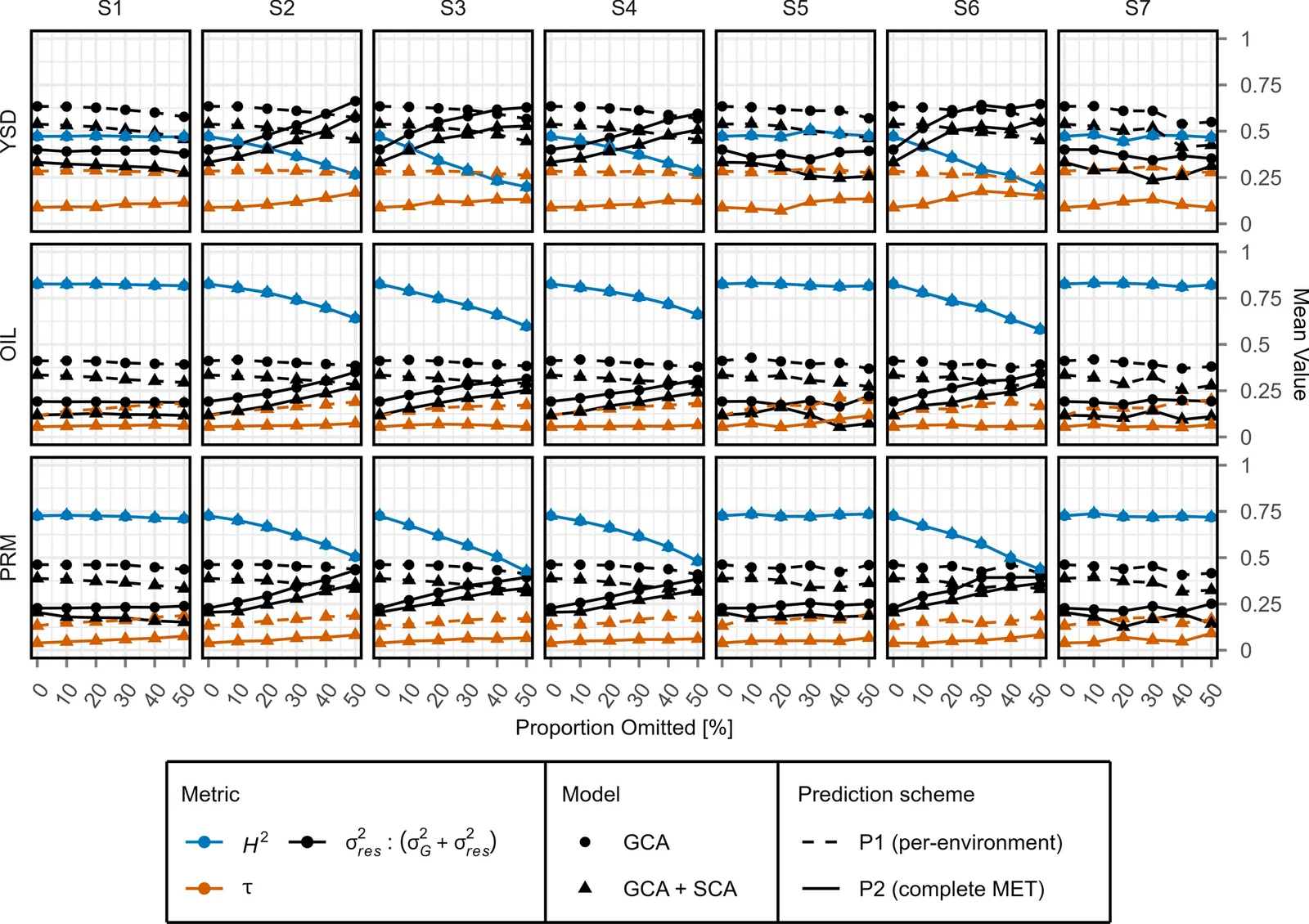
Mean estimates of broad-sense heritability (${H}^{2}$), relative contributions of specific combining ability (SCA) variance to total genetic variance (τ), and proportions of residual variance to the total variance (${\sigma}_{\mathit{res}}^{2} :({\sigma}_{\mathit{G}}^{2}+{\sigma}_{\mathit{res}}^{2})$) across seven omission scenarios (S1-S7) for grain yield (YSD), oil content (OIL), and protein content in meal (PRM). Results for relative contributions of SCA variance are separated for the prediction schemes (P1 = per-environment, P2 = complete-MET) and are, as for broad-sense heritability, mean values over all iterations and multi-environment field trials (MET). Variance components were estimated using pedigree-genomic relationship matrices (${G}^{(H)}$) and complete sets of hybrids

Throughout, predictions for OIL and PRM showed higher mean prediction accuracy and Cohen’s *κ* than those for YSD, indicating greater robustness to data omission. Those differences in the robustness were reflected in their underlying variance structure, as OIL (ca. 0.83) and PRM (ca. 0.73) exhibited higher mean ${H}^{2}$ compared to YSD (ca. 0.47) and smaller proportions of the residual variance to the total variance (${\sigma}_{\mathit{res}}^{2} :({\sigma}_{\mathit{G}}^{2}+{\sigma}_{\mathit{res}}^{2})$) across omission scenarios (Fig. 5). Under balanced omission scenarios (S1, S5, and S7), ${H}^{2}$ estimates remained largely stable as omission increased, whereas unbalanced scenarios (S2, S3, S4, and S6) resulted in declining ${H}^{2}$ estimates, with strongest reductions observed for YSD and PRM.

Contrary with the patterns of ${H}^{2}$ estimates, $\sigma_{{{\mathit{res}}}}^{2} :\left( {\sigma_{{\mathit{G}}}^{2} + \sigma_{{{\mathit{res}}}}^{2} } \right)$ under the complete-MET prediction schemes (P2) increased primarily under unbalanced omission scenarios and was highest for YSD (Fig. 5). Under balanced omission (S1, S5, and S7), $\sigma_{{{\mathit{res}}}}^{2} :\left( {\sigma_{{\mathit{G}}}^{2} + \sigma_{{{\mathit{res}}}}^{2} } \right)$ in P2 showed no consistent trend across traits and remained largely stable across omission rates. Under per-environment predictions (P1), $\sigma_{{{\mathit{res}}}}^{2} :\left( {\sigma_{{\mathit{G}}}^{2} + \sigma_{{{\mathit{res}}}}^{2} } \right)$ for the GCA model remained comparatively stable for OIL and PRM across all omission scenarios, while YSD exhibited a modest but consistent decline with increased omission rate. For the GCA + SCA model in P1, reductions in $\sigma_{{{\mathit{res}}}}^{2} :\left( {\sigma_{{\mathit{G}}}^{2} + \sigma_{{{\mathit{res}}}}^{2} } \right)$ under severe data omission were similar across scenarios and traits. Overall, across traits, omission scenarios, and prediction schemes, the proportions of $\sigma_{{{\mathit{res}}}}^{2} :\left( {\sigma_{{\mathit{G}}}^{2} + \sigma_{{{\mathit{res}}}}^{2} } \right)$ were consistently lower for the GCA + SCA model than for the GCA-only model.

The contribution of SCA variance to the total genetic variance (τ) after Eq. 5 was small for YSD and lowest for OIL and PRM (Fig. 5). Values of τ showed minor increases at higher omission rates and were consistently lower under the complete-MET prediction scheme (P2) (0.09, 0.05, and 0.04) than under the per-environment predictions (P1) (0.28, 0.11, and 0.13, for YSD, OIL, PRM, respectively).

**Fig. 6:**
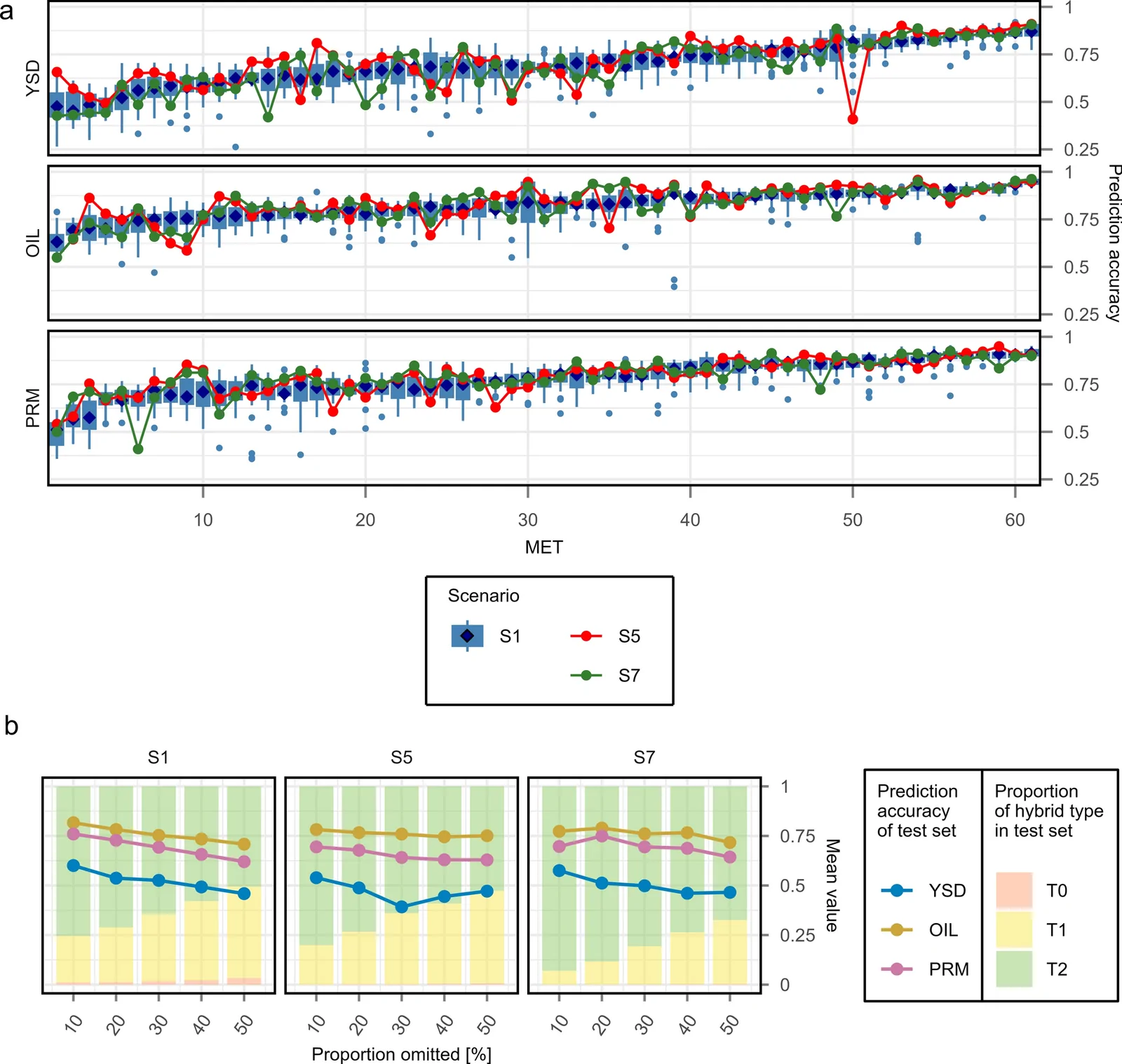
Panel **a** shows the distribution of prediction accuracies under the balanced omission scenarios S1 (random uniform omission), S5 (relationship-based uniform omission) and S7 (uniform omission based on genetic algorithm) for grain yield (YSD), oil content (OIL), and protein content in meal (PRM). Results are shown for complete-MET predictions (P2) using the pedigree-genomic relationship matrices (${G}^{(H)}$) and the GCA + SCA model at 50% omission rate. For each multi-environment field trial (MET), S1 boxplots summarize 25 random iterations, whereas S5 and S7 represent single realizations. METs are ordered by increasing prediction accuracy. Panel **b** displays the proportions of hybrid types (T2 = both parents in training set, T1 = one parent represented in the training set, T0 = no parent in the training set) in the test set and the corresponding prediction accuracies of the test set for S1, S5 and S7 across omission rates from 10 to 50%. Prediction accuracies and proportions of hybrid types represent means across all runs (for S1) and METs

Given that balanced omission scenarios constantly achieved the highest prediction performance, subsequent results are restricted to a direct comparison of scenarios S1, S5, and S7. Those three omission scenarios apply uniform omission across environments and therefore preserved balance in the training data, allowing a targeted comparison under the best-performing prediction setting (P2, GCA + SCA, ${G}^{(H)}$) (Fig. 6a). Across METs and traits, S5 achieved prediction accuracies that were slightly higher than those obtained under S1, especially in METs with tendentially lower mean prediction performance and for more complex traits. Importantly, realizations under S5 and S7 exhibited reduced variability in prediction accuracy across METs, whereas S1 showed greater dispersion across the 25 random iterations. Overall, S1, S5, and S7 maintained high prediction performance with balanced data reduction, while S5 showed marginally higher (+ 0.02) prediction accuracy and S7 no increase in prediction accuracy across METs (Fig. 6) compared to S1.

To assess the impact of omission scenarios on prediction accuracy for untested hybrids, prediction accuracies were extracted for test set alongside the overall prediction accuracy. Test set prediction accuracies were uniformly lower (Fig. 6b) than for whole sets of hybrids of METs (Fig. 3b) and similarly exhibited declining trends with increasing omission rates. The higher heritable traits Oil and PRM (Fig. 5) maintained superior test set prediction accuracies compared to YSD (Fig. 6b). Critically, no substantial differences in test set prediction accuracy emerged among S1, S5, and S7, with initial values at 10% omission rate of 0.55, 0.79, and 0.71 for YSD, OIL, and PRM, respectively, decreasing to 0.46, 0.73, and 0.63 at 50% omission rate. As omission rates increased from 10 to 50%, the proportion of omitted hybrids in the test set with both parental lines represented in the training set (T2) declined systematically across the omission scenarios S1, S5, and S7, with reductions primarily compensated by an increased proportions of hybrids with one parent contained in the training set (T1) (Fig. 6b). Only test sets under S1 comprised a minor proportion of T0 hybrids (no parent represented in the training set). The proportions of hybrid types in the test set were invariant across traits. Compared to the similar hybrid type distribution patterns observed for S1 and S5 (51% T2, 47% T1, and 2% T0 at 50% mission rate), S7 consistently exhibited larger T2 proportions (68% T2, 32% T1, and 0% T0 at 50% omission rate), indicating that the genetic algorithm more effectively maintained parental line representation for hybrids in the test (Fig. 6b).

## Discussion

The systematic decline in both prediction accuracy and selection consistency with increasing proportion of omitted phenotypic records highlights the sensitivity of genomic prediction to data restriction (Fig. 3). Notably, our evaluation was conducted across 61 METs that reflect typical variation in breeding data regarding hybrid set composition and environments, which strengthens the robustness of our observations. While reductions in prediction accuracy with decreasing training population size are well documented (Isidro et al. 2015; Zhang et al. 2017; Akdemir and Isidro-Sánchez 2019; Edwards et al. 2019; Kaler et al. 2022), the moderate correlation between prediction accuracy and selection consistency (Cohen’s *κ*) in our study indicates that both evaluation metrics do not scale linearly. Consequently, evaluations of prediction performance should rely on complementary interpretations rather than on prediction accuracy alone (Heilmann et al. 2024). The high prediction performance observed in the absence of data omission supports a biologically meaningful baseline and confirms the strong suitability of genomic prediction for the present dataset. This indicates that the available phenotypic, genotypic, and pedigree information captured a substantial proportion of the underlying genetic signal.

Across omission scenarios, models based on combined pedigree–genomic relationship matrices (${G}^{(H)}$) consistently showed similar prediction accuracy compared to models relying exclusively on genomic prediction matrices (${G}^{(G)}$) (Fig. 3). An advantage of higher and more stable prediction accuracies was also observed by Habier et al. (2007), Goddard et al. (2011), and Sood et al. (2020). Velazco et al. (2019) indicated an optimization of prediction performance especially for traits with lower heritability, such as YSD, while Ashraf et al. (2016) and Martini et al. (2018) reported benefits from including non-genotyped individuals using ${G}^{(H)}$. In our study, we specifically added 440 non-genotyped parental lines, thereby increasing the training population size and the effective information content of the training population. Although ${G}^{(H)}$ includes more individuals than ${G}^{(G)}$, both matrices are conceptually comparable, as each fully exploits the information available: genomic data for ${G}^{(G)}$, and pedigree plus genomic data for ${G}^{(H)}$. As only marginal differences were observed when training sets of ${G}^{(H)}$ were matched with ${G}^{(G)}$, the results in Fig. 4 demonstrate a key practical benefit of being able to include 440 non-genotyped parental lines in the training populations, thereby expanding predictions to a larger set of hybrids without compromising prediction accuracy. Critically, the addition of pedigree information did not decrease prediction accuracy in our study but maintained prediction performance while allowing evaluations of a broader set of hybrids.

Furthermore, our results suggest a moderate but systematic impact of the omission scenario on the prediction accuracy and selection consistency in genomic predictions (Fig. 3). The lower performance of S3 and S6 indicates that retaining complete datasets in internal environments while focussing on uniform omissions in external environments can reduce the effective representation of hybrids, particular for environmentally more sensitive traits, such as YSD. Similar findings have been reported by Jarquin et al. (2020) and Crespo-Herrera et al. (2021), who showed that increasing genotype overlap across environments improves prediction accuracy. In contrast, independent omission across environments (S2 and S4) maintained broader environmental coverage for individual hybrids, resulting in moderately improved prediction performance, despite reduced overlap within individual environments, consistent with observations by Lell et al. (2021). Likewise, Crespo-Herrera et al. (2021) and Gill et al. (2021) demonstrated that prediction accuracy can be maintained under sparse testing designs when genotype representation is sufficiently distributed across environments. Overall, retaining complete phenotypic data in a subset of environments (S3, S4, and S6) showed no clear advantage for genomic prediction performance, in agreement with the findings of Lell et al. (2021) for genomic prediction in sparsely tested hybrid wheat.

Among the omission scenarios, uniform omission across all environments (S1, S5, and S7) improved prediction performance compared to the four other omission scenarios by preserving balance between hybrids and environments, thereby stabilizing model estimation under reduced training set size (Fig. 3). The superiority of relationship-based omission (S5) further demonstrates that incorporating genetic relatedness into the omission process provides an additional, albeit modest, advantage over random uniform omission (S1). This improvement likely reflects a more efficient retention of genetic information within the training set, consistent with previous findings reported by Atanda et al. (2021), and demonstrates the scope for action considering the structural data limitations and genetic relatedness typical of datasets in applied plant breeding.

Although omission strategy and model configurations exerted clear influences on prediction performance, no single omission rate can be universally recommended based solely on breeding progress. Increasing levels of data omission inevitably reduced prediction accuracy and selection consistency. However, moderate reductions in phenotypic testing may be justified when balanced against the costs of field experiments and genotyping (Heslot et al. 2015). In practical breeding, the objective is to optimize genetic gain relative to resource investment rather than maximizing prediction accuracy per se (Cobb et al. 2019; Vieira et al. 2025). Accordingly, balanced and relationship-based omission strategies offer a pragmatic compromise by maintaining robust prediction performance under reduced testing intensity.

Beyond the omission scenario itself, prediction performance was also influenced by whether predictions were performed per-environment (P1) or complete-MET (P2). Across all traits and omission scenarios (S1-S7), both prediction accuracy (+ 0.05, + 0.03, and + 0.03) and Cohen’s *κ* (+ 0.06, + 0.07, and + 0.05) were consistently higher for P2 than for P1, respectively, for YSD, OIL, and PRM (Fig. 3). The advantage of P2 further increased with higher omission levels in scenarios S2-S5, underscoring that aggregating phenotypic data across environments prior to prediction provides greater stability against data reduction than an aggregation conducted after environment-specific predictions. This is consistent with findings from Terraillon et al. (2022), who reported genomic predictions can remain accurate under low-intensity METs, emphasizing that effective structuring of available information, whether across environments or via genetic pseudo-replication, strengthens prediction accuracy. Comparable improvements of multi-environment over single-environment predictions have been reported in wheat (Cuevas et al. 2016; Tomar et al. 2021; Lopez-Cruz and de los Campos 2025), maize (Bandeira e Sousa et al. 2017), and field peas (Saludares et al. 2024), supporting the generality of this observation across species. Together, these results indicate that genomic predictions leveraging phenotypic data across environments are more effective for capturing stable genetic effects and thereby enhance the robustness of hybrid performance predictions under conditions of data reduction.

The choice of statistical model, specifically whether to include SCA effects in additions to GCA effects, had a modest impact on prediction performance across omission scenarios or prediction schemes. On average, the inclusion of SCA effects increased prediction accuracies by + 0.05, + 0.03, and + 0.03, and Cohen’s *κ* by + 0.07, + 0.05, and + 0.05, for YSD, OIL, and PRM, respectively (Fig. 3). These consistent improvements with SCA inclusion, particular for traits like YSD, OIL, and PRM, are align with other results in *B. napus* (Azizinia 2012; Rameeh 2012; Dezfouli et al. 2019; Rao et al. 2025), in sorghum (Fonseca et al. 2021), or simulated maize and wheat hybrids (Melchinger and Frisch 2023). In the present study, the contribution of SCA variance to total genetic variance (τ) was small across traits and lowest for OIL and PRM (Fig. 5), indicating that the observed gains from SCA inclusion did not primarily arise from large SCA variance components. As demonstrated by González-Diéguez et al. (2021), it is important to note that SCA terms constructed via element-wise multiplications of parental relationship matrices specifically capture additive × additive epistatic variation between heterotic groups. In practical breeding datasets, SCA may capture not only additive × additive epistasis but also non-additive signals due to confounding. The benefits of including SCA were most evident at low omission rates, suggesting that these effects primarily improve model fit for training data. Especially at 0% omission rate, prediction accuracies should be interpreted with caution, as they may partly reflect model overfitting to training data rather than performance of the inclusion of true SCA effects (Fig. 3). However, as the objective of this study was not to compare predictive accuracy for untested hybrids but model robustness under different phenotypic data reductions, this limitation does not affect the relative comparison of omission scenarios.

Our observed τ values contrast with findings by Heilmann et al. (2023), who reported substantially higher τ values (0.67, 0.51, and 0.28) in three *B. napus* populations, where τ was identified as a major determinant of overall prediction accuracy. Notably, Heilmann et al. (2023) also observed markedly reduced prediction accuracy when models were restricted to GCA effects, particular in populations with high τ, and demonstrated that stacked ensembles approaches were effective in exploiting strong SCA signals. Together, these findings indicate that the benefit of including SCA effects depends on both the proportion of SCA variance and the model framework applied. Populations with larger SCA variance can derive substantial benefit from more flexible modeling approaches that explicitly account for SCA. Conversely, when SCA contributions are small, as observed in this study, increased model complexity yields only modest improvements, consistent with reports in sunflower (Mangin et al. 2017), maize (Kadam and Lorenz 2019), potato (Adams et al. 2023).

Trait-specific differences in robustness to data omission were evident, with OIL and PRM showing greater resilience than YSD across prediction scheme and models (Fig. 3). This pattern is accordant with their generally higher heritability and lower residual variance (Fig. 5), which facilitate more reliable genomic predictions, whereas the lower heritability and more complex genetic architecture of YSD limit prediction accuracy. Comparable results in commercial wheat breeding indicated that phenotypic data quality, rather than population size, limits genomic prediction for complex traits (Thomsen et al. 2026). Omission scenarios further modified these effects, as balanced omission across environments (S1, S5 and S7) largely preserved heritability, whereas unbalanced omission resulted in declining heritability, particularly for YSD (Fig. 5). Robust variance estimation occurs under missing data when losses are random or evenly distributed, keeping parameter estimates stable; in contrast, structured or biased omission distort variance components and reduced heritability estimates (Lourenço et al. 2020). In line with this interpretation, previous studies reported higher prediction accuracies for seed quality traits in *B. napus* (Jan et al. 2016; Zou et al. 2016) compared to yield-related traits (Jan et al. 2016; Fonseca et al. 2021; Crossa et al. 2025), reflecting the higher heritability and lower environmental sensitivity of the former.

The direct comparison of the best-performing and balanced omission strategies highlights a consistent, albeit moderate, advantage of relationship-based omission (S5) over random (S1) or genetic algorithm (S7) omission. While these three scenarios kept data balanced and parameters for variance estimation stable, S5 yielded slightly higher mean prediction accuracies, especially in METs with lower prediction performance, and, more importantly, reduced variability in prediction outcomes (Fig. 6a). This increased stability indicates that selecting hybrids for omission based on genetic and pedigree relatedness more effectively preserves genetic connection between training set and test set than purely random omission and was proven to enhance prediction accuracy in other studies (Lorenz and Smith 2015; Zhang et al. 2018).

Aligned with that, S5 retains T2 hybrids in the test set and prioritizes the reduction of T0 hybrids. While Technow et al. (2014) established that predicting T2 hybrids consistently achieved the highest prediction accuracies, our study revealed that S7, despite maintaining 68% T2 hybrids in the test set at 50% omission rate (compared to 51% for S1 and S5) (Fig. 6b), did not exhibit superior prediction accuracy, confirming that test set hybrid composition alone is not the driver of prediction performance, but training set connectivity. Furthermore, under random omission (S1), prediction performance is additionally subject to stochastic variability, implying an increased risk of suboptimal outcomes in practice, where METs are typically conducted only once. The advantage of S5 was particularly evident in METs with lower prediction accuracy (Fig. 6a), suggesting that relationship-informed partitioning of individuals into training set and test set provides additional superiority under less favorable data conditions. The consistently lower test set prediction accuracies (Fig. 6b) relative to overall prediction accuracies (Fig. 3b) confirm the absence of overfitting in balanced omission scenarios. Notably, the similar test set prediction accuracies of random omissions (S1) and relationship-informed omission (S5) indicate that the latter do not artificially inflate prediction accuracy but provide a more stringent assessment of model generalization (Fig. 6b). These test set prediction accuracies evaluate untested hybrids at 10–50% omission rates and align with the expectation that true prediction performance should be conservative when evaluated on independent test sets (de los Campos et al. 2013). Overall, our findings support relationship-informed, balanced omission (S5) as a robust strategy for sustaining genomic predictions.

## Conclusion

This study demonstrates that genomic prediction performance under data reduction is strongly shaped by omission strategy, prediction scheme, relationship modeling, and trait architecture. Our results indicate that breeders can actively influence prediction performance through informed design choices and provide scope for adaptation and improvement. The use of ${G}^{(H)}$ further allows to leverage both genotyped and non-genotyped parental information, increasing the return on genotyping investments. Nevertheless, strong environmental effects and trait-specific genetic architecture necessitate cautious interpretation. Overall, tailored genomic prediction strategies offer substantial potential to optimize genetic gain under practical resource constrains, while continues validation across populations and crops remains essential.

Across all traits, the most robust and consistent results were achieved using a combination of balanced omission across environments, complete-MET prediction (P2) in models with the inclusion of both GCA and SCA effects and ${G}^{(H)}$. In particular, the balanced relationship-based omission strategy (S5) consistently outperformed random and genetic-based uniform unbalanced omission by delivering higher prediction accuracies. Therefore, we propose this relationship-informed, balanced subsampling method as an approach that reduces phenotyping efforts and warrants further evaluation in future breeding studies.

## Supplementary Information

Below is the link to the electronic supplementary material.

Supplementary file1 (DOCX 345 KB)

## Data Availability

The datasets analyzed during the current study are available from the corresponding author upon reasonable request.
