# Supplementary material for: Improving hybrid breeding efficiency in winter oilseed rape under sparse multi-environment testing
Source: Theor Appl Genet. 2026 Aug 12;139(9):227. doi: 10.1007/s00122-026-05341-3 (PMC13469503; doi:10.1007/s00122-026-05341-3)
Supplement: Supplementary file 1 [file 122_2026_5341_MOESM1_ESM.docx]

Improving Hybrid Breeding Efficiency
in Winter Oilseed Rape
under Sparse Multi-Environment Testing

Lars Erik Thomsen^ab*^; ORCiD: 0009-0003-7405-5338;
E-mail: lars.e.thomsen@agrar.uni-giessen.de; Tel.: +49 641 9937547.

Matthias Frisch^a^; ORCiD: 0000-0003-4234-8972;
E-mail: matthias.frisch@uni-giessen.de; Tel.: +49 641 9937540.

Christian Flachenecker^c^;
E-mail: c.flachenecker@npz.de; Tel.: +49 4351 736-309.

Amine Abbadi^b^; ORCiD: 0000-0002-2389-978X;
E-mail: a.abbadi@npz-innovation.de; Tel.: +49 4351 736-164.

Tobias Kox^c^;
E-mail: t.kox@npz.de; Tel.: +49 4351 736-235.

Cyriakus Kulle^b^;
E-mail: c.kulle@npz-innovation.de; Tel.: +49 4351 736-206.

Carola Zenke-Philippi^a^; ORCiD: 0000-0002-4484-8746;
E-mail: carola.zenke-philippi@uni-giessen.de; Tel.: +49 641 9937555.

^a^ Department of Plant Breeding, Justus-Liebig-University Giessen, Heinrich-Buff-Ring 26, 35392 Giessen, Germany.

^b^ NPZ Innovation GmbH, Hohenlieth Hof 1, 24363 Holtsee, Germany.

^c^ Norddeutsche Pflanzenzucht Hans-Georg Lembke KG, Hohenlieth Hof 1, 24363 Holtsee, Germany.

* Corresponding author. E-mail: lars.e.thomsen@agrar.uni-giessen.de (L. Thomsen).

# 1 Supplementary Information (SI)

**SI 1** Data availability across locations and 61 multi-environment field trials (MET) per year

**SI 2** First two dimensions of a principal coordinate analysis (PCoA) for 72 hybrids from an exemplary multi-environment field trial, illustrating (**a**) the relationship-based deterministic omission method for iterative division into training and test set. Red numbers denote the omission order of non-check hybrids assigned to the test set, (**b**) the genetic algorithm division into to training and test set. Red dots show hybrids designated to the test set, black dots represent check hybrids, and blue dots indicate retained non-check hybrids used for model training

**SI 3** First, second and third principal coordinates (PCo 1, PCo 2, PCo 3) for 1,284 parental lines with genotypic data (a-c), and (d) cumulative variance explained by the first 100 principal components
